# Editorial: Biology, systematics, and evolution of ferns and lycophytes in the omics era

**DOI:** 10.3389/fpls.2023.1146829

**Published:** 2023-02-23

**Authors:** Alexandre Salino, Germinal Rouhan, Li-Yaung Kuo, Thaís Elias Almeida

**Affiliations:** ^1^ Programa de Pós-Graduação em Biologia Vegetal, Universidade Federal de Minas Gerais, Belo Horizonte, Brazil; ^2^ Institut de Systématique, Evolution, Biodiversité (ISYEB), Muséum national d’Histoire naturelle, Sorbonne Université, EPHE, UA, CNRS, Paris, France; ^3^ Institute of Molecular & Cellular Biology, National Tsing Hua University, Hsinchu, Taiwan; ^4^ Universidade Federal de Pernambuco, Departamento de Botânica, Centro de Biociências, Recife, Brazil

**Keywords:** genome evolution, hybridization, phylogenetics, plastome, transcriptome

Ferns and lycophytes are distinct evolutionary lineages of vascular plants, with ferns being the sister group to seed plants. The last common ancestor of seed plants, ferns, and lycophytes is the ancestral vascular plant. Historically, both lineages have been studied together and treated as the paraphyletic group ‘pteridophytes’, mainly because both lineages are spore-bearing, and share many other biological features, such as the overall life cycle (PPG I, 2016). The publication of the first complete genome of a fern took longer than other groups of embryophytes (Li et al., 2018). The first two reported genomes, from the heterosporous and aquatic *Azolla* and *Salvinia*, are atypical among ferns as they are less than tenfold smaller than the average fern genome (Li et al., 2018). The first homosporous fern genomes were only recently published (Fang et al., 2022; Huang et al., 2022; Marchant et al., 2022; Rahmatpour et al., 2023). Although ferns and lycophytes are less speciose than flowering plants in terms of extant diversity, their biology is crucial for understanding land plant evolution, diversification, and origins. Inferring evolutionary processes and patterns can be facilitated by access to all kinds of omics data (e.g. genomics, metagenomics, transcriptomics, proteomics). Despite the availability of high-throughput sequencing data, publications on ferns and lycophytes at the omics scale are still few relative to their importance for understanding the diversity and biology of vascular plants. Six articles in this Research Topic examined evolutionary questions such as whole genome duplication, gene retention, structural variation in plastomes, conflicts in plastid phylogenomics, species delimitation, hybridization, and introgression, as well as a new pipeline for continuously and updated Fern Tree of Life (FTOL) generation.

For about three decades, insights from molecular data have contributed to revolutionizing our understanding of fern and lycophyte systematics and evolution (e.g. Raubeson and Jansen, 1992; Pryer et al., 1995; Pryer et al., 2001; Schneider et al., 2004; Schuettpelz and Pryer, 2007; Lehtonen, 2011; Testo and Sundue, 2016). Nitta et al. developed an outstanding automated, reproducible, and open pipeline to generate a continuously updated fern tree of life from DNA data available in GenBank named by the authors as FTOL (Fern Tree of Life). They combined published data from whole plastomes and commonly sequenced plastid regions to generate a species-level phylogeny with 5,582 species. Nitta et al. also used the most complete list ever assembled of 51 fern fossil constraints and estimated ages older than previous studies for families and broader clades. The authors provide the list of fossils, a taxonomic database, and R packages *via* a web portal (https://fernphy.github.io). All in all, FTOL is destined to become a landmark tool for all research on this key group of plants over a wide range of taxonomic scales.

So far, most of the studies are based exclusively on plastid regions and are used to reconstruct phylogenetic relationships, patterns, and processes of biogeographic history above and at the species level. Inferring phylogenies from plastid sequences remains a powerful tool, such as for barcoding (Hollingsworth et al., 2016). High copy numbers of plastid genomes exist in cells, their structure is highly conserved, and they are thus easily amplified. It has been hypothesized to evolve as a single locus (Léveillé-Bourret et al., 2017), but for some groups, congruence tests have shown that this is not always the case (e.g. Gonçalves et al., 2019). Focusing on fern plastomes, Du et al. investigated the structural evolution of chloroplasts in deep nodes of the fern tree of life, with a sampling including all recognized fern families and orders (PPG I, 2016) and 127 plastomes. The authors were able to map several structural synapomorphies, including inversions, changes in inverted repeats (IR) boundaries, and gene losses, and have shown that untypical structures such as loss of IR, or the presence of DR (direct repeat) found in other lineages of land plants, are not present in the ferns chloroplast. The authors used their dataset to reconstruct a phylogenetic inference and to identify an intermediate plastomic structure, supporting evidence that the relationship among some orders, such as the early-diverging leptosporangiate lineages, Gleicheniales, Hymenophyllales, and Matoniales (Dipteridaceae + Matoniaceae) is still controversial.

Focusing on a recalcitrant node in the fern tree of life, Wang et al. tested for conflicts and systematic errors in plastome-based phylogenetic inferences. The authors used 30 different datasets built on different strategies using coding and noncoding regions of plastomes from 42 species, independently and combined, using a maximum likelihood and a coalescent tree-based method, all compared to an aminoacid-based tree, to solve the phylogenetic positioning of and relationships within Dennstaedtiaceae. They found that addressing systematic errors helps to reduce conflict, but incongruences were inherently present and should be taken into account when using plastomes for phylogenetic inferences.

From a different perspective, Pelosi et al. used available transcriptomic data covering a broad sampling of families to investigate the backbone of fern phylogeny. The overall recovered topologies based on their nuclear dataset are consistent with most published phylogenetic inferences based on plastid and nuclear data (e.g. Qi et al., 2018; Shen et al., 2018); however, there are some recalcitrant nodes, including the sister group to leptosporangiates and eupolypods, the monophyly of Gleicheniales + Hymenophyllales (disagreeing with the plastome results by Du et al.), and the positioning of Aspleniaceae. The authors also focused on whole genome duplication (WGD) events across the fern phylogeny, finding deep and shallow WGD events along the fern tree, with low gene retention as found in other groups of plants for ancient events of duplication.

Moving from the backbone to the specific clades of the fern tree of life, Ke et al. reconstructed the phylogenetic relationships of the Schizaeaceae family, using the broadest sampling to date, based on three plastid regions and plastomes of selected taxa. The authors traced the evolutionary history of the gene losses in the plastid genome associated with the evolution of gametophytic mycoheterotrophy, presenting a novel phylogenetic classification for the family with the recognition of a third genus — *Microschizaea* — segregated from *Schizaea* sensu PPG I (2016). A newly described species of *Schizaea* is presented.

Focusing on species-level evolution, Petlewski et al. present a phylogenetic analysis of lycophyte *Dendrolycopodium* (Lycopodiaceae), focusing on species delimitation and hybridization and using restriction-site associated DNA sequencing (RADseq) and a draft genome assembly. The authors found that *Dendrolycopodium* can be divided into four clades that largely correspond to the described taxa, although the status of the various Asian species remains uncertain. Petlewski et al. confirm evidence of interspecific hybridization and the occurrence of introgression in the group.

Omics data reveal new insights into ferns and lycophytes, as well as how these tools and data can help us to better understand the evolution of these lineages. In the near future, with the ease of access to tools and the low cost of sequencing, we expect to see more and more studies in the Omics and integrative Multi-Omics that focus on ferns and lycophytes. Species evolution, reticulation (e.g. hybridization), polyploidy, population genomics, biogeography, cytogenomics, and evolutionary developmental biology are some of the areas that may benefit from such endeavors.

## Author contributions

AS, GR, L-YK, and TA drafted the manuscript. All authors contributed to the article and approved the submitted version.
